# A Polarized Raman Spectroscopic Method for Advanced Analyses of the Osteon Lamellar Structure of Human Bone

**DOI:** 10.3390/mps5030041

**Published:** 2022-05-20

**Authors:** Giuseppe Pezzotti, Eiji Ishimura, Ryosuke Inai, Wenliang Zhu, Taigi Honma, Nobuhiko Sugano, Wataru Ando, Ugo Pazzaglia, Elia Marin

**Affiliations:** 1 Ceramic Physics Laboratory, Kyoto Institute of Technology, Sakyo-ku, Matsugasaki, Kyoto 606-8585, Japan; eijiishimura4474@gmail.com (E.I.); bluenobody13@gmail.com (R.I.); wlzhu@kit.ac.jp (W.Z.); patagonia.th.132132@gmail.com (T.H.); 2 Department of Dental Medicine, Graduate School of Medical Science, Kyoto Prefectural University of Medicine, Kamigyo-ku, Kyoto 602-8566, Japan; 3 Department of Immunology, Graduate School of Medical Science, Kyoto Prefectural University of Medicine Kami-gyo-ku, 465 Kajii-cho, Kawaramachi dori, Kyoto 602-0841, Japan; 4 The Center for Advanced Medical Engineering and Informatics, Osaka University, Osaka 565-0871, Japan; 5 Department of Orthopedic Surgery, Tokyo Medical University, 6-7-1 Nishi-Shinjuku, Shinjuku-ku, Tokyo 565-0871, Japan; 6 Kyoto Municipal Science Center for Youth, Kyoto City Board of Education, Fushimi-ku, Fukakusa, Kyoto 612-1601, Japan; 7 Department of Orthopaedic Medical Engineering, Osaka University Graduate School of Medicine, Suita, Osaka 565-0871, Japan; n-sugano@umin.net (N.S.); w-ando@umin.ac.jp (W.A.); 8 Department of Medical and Surgical Specialties, Radiological Sciences & Public Health, University of Brescia, 11 Viale Europa, 25123 Brescia, Italy; ugo.pazzaglia@unibs.it

**Keywords:** Raman, osteon, human bone, structure, polarized spectroscopy

## Abstract

Raman spectroscopy has recently been used for quantitative analyses of cortical bone tissue and related materials, such as dentin and enamel. While those analyses have proven useful as potential diagnostic tools, the Raman spectrum of bone encrypts a wealth of additional molecular scale details about structure and crystal arrangement, which are yet to be unfolded. Such details directly link to both bone physiology and pathology. In this work, a triple monochromator spectrometer with high spectral resolution, employed in polarized light configurations, was used to extract quantitative details about the preferential crystallographic orientation of apatite and collagen components in a human proximal femoral cortical bone sample. This body of information was then used to model the bone structure at the nanometric scale through a methodology that could be key in assessments of bone structure in health and disease.

## 1. Introduction

While crystallographic arrangement, chemical composition, and micromechanical response have long been recognized as the main factors determining the physiological response of bone, the analytical methodologies presently available to investigate its structure at the microscopic scale usually fail in a concurrent and exhaustive description of those factors. As previously shown for enamel [1], dentin [2], and apatite [3], quantitative parameters derived from Raman spectra correlate with crystallographic, chemical, and micromechanical characteristics of bone in a single, fast, and non-destructive experiment.

In recent years, Raman spectroscopy has been used as a complementary diagnostic tool for bone quality and bone-related diseases. Morris and Mandair [4] produced the first comprehensive review on the Raman assessment of bone quality, back in 2011. Raman spectra have been reported for human and animal bone as a function of age, biomechanical status, pathology, and other quality parameters. The cited literature supported the use of the mineral-to-matrix ratio, carbonate-to-phosphate ratio, and mineral crystallinity as measures of bone quality, but the authors also noted discrepancies in the methodologies and even different interpretations for the physical meaning of a few Raman bands. The same research team was later able to correlate the Raman spectra of bone with mechanical properties, in particular by considering the ratio between mineral- and matrix-related bands [5]. It was also observed that both the orientation of the mineral crystallites with respect to the collagen fibril axis [6] and the amount of cross-linking of collagen [7] play a crucial role in determining the mechanical strength of bone, and both parameters could be estimated by using Raman spectroscopy. Quality of bone and, in particular, the mineral-to-matrix ratio is altered dramatically in bone-related diseases such as osteoporosis [8], osteonecrosis [9] or osteogenesis imperfecta [10]. In those reports, the spectroscopic analysis was subsequent to the diagnosis, making Raman spectroscopy a complementary diagnostic tool at best.

The three-dimensional arrangement of hydroxyapatite crystals, their chemical structure and alterations in presence of bone disease, and the micromechanical response to external stress of their structural ensemble can be effectively analyzed, provided that suitable algorithms are built up to extract such information out of a Raman spectrum possessing a sufficient resolution [1]. This could be done through coupling a polarized Raman algorithm developed from the selection rules of hexagonal hydroxyapatite to the statistical orientation distribution function (ODF) formalism. Osteonal lamellae are considered as the primary building blocks of lamellar bone. They have a parallel-layered structure (i.e., with a thickness typically ranging between 3 and 7 μm), and a composite of collagen fibrils and mineral apatite platelets, which are, in turn, embedded in a mineralized extra-fibrillar matrix [11,12,13,14].

In this paper, we specifically analyze the anisotropic intensity of the Raman bands of bony hydroxyapatite as a function of orientation of incident laser polarization and relate it to the average orientation of the hexagonal (nano-sized) hydroxyapatite crystallites and to their degree of alignment in human osteonal lamellae. A comparison is also carried out with the major fibrillar organization patterns identified by other authors for the collagen component [11]. The results obtained here at the osteon sites of human lamellar bone vividly show the versatility of the analytical Raman approach in the crystallography of biogenic materials and specifically demonstrate its capability in providing three-dimensional structural information in the hierarchical structure of bone. It is believed that using Raman spectroscopy to derive a set of structural, micromechanical, and chemical nature could contribute to build up a more comprehensive view of bone tissue at the molecular level.

## 2. Materials and Methods

A human proximal femoral bone tissue sample was obtained from the Department of Medical and Surgical Specialties, Radiological Sciences, and Public Health, University of Brescia. The bone specimen was obtained from amputation in a 32-year-old male subject who underwent severe traumatic limb injuries. The patient gave its consent for a segment of the amputated bone to be used for scientific purposes, and the Council of the Department Medical and Surgical Specialties, Radiological Sciences, and Public Health of the University of Brescia approved the study protocol. The specimen was stored at 4 °C in neutral formaldehyde solution (10%) until further processed. The sample was cut with a low-speed, circular, diamond-coated saw (Remet, Casalecchio di Reno, Bologna, Italy) with the shape of parallelepipeds size about 3 × 4 × 15 mm with the longer side corresponding to the diaphyseal long axis.

Raman spectra were collected using a spectroscope (T-64000, Horiba/Jobin-Yvon, Kyoto, Japan) operating in microscopic confocal mode with 10× or 100× optical lenses. A triple monochromator was used for high-resolution spectral acquisitions. A spectral resolution of 0.1 cm^−1^ could be achieved with implementing the high resolution of the spectrometer with the systematic collection of a signal from a neon lamp as an internal reference to eliminate possible wavenumber fluctuation at each measurement. A 488 nm Ar-ion laser (Stabilite 2017, Spectra Physics, Mountain View, CA, USA) was used as an excitation source and applied with a power of 10 mW. The Raman light was diffracted into a monochromator connected with an air-cooled 1024 × 256 pixels charge-coupled device (CCD) detector (CCD-3500V, Horiba Ltd., Kyoto, Japan). The acquisition time for one spectrum was 30 s. The collected spectra were analyzed after being deconvoluted into Lorentzian–Gaussian sub-bands using commercially available software (LabSpec 4.02, Horiba/Jobin-Yvon, Kyoto, Japan). All spectra were represented in Origin 6.0 (Microcal Software Inc., Northampton, MA, USA), subtracted of their background, and then smoothed with the FFT Filter tool.

**Figure 1:**
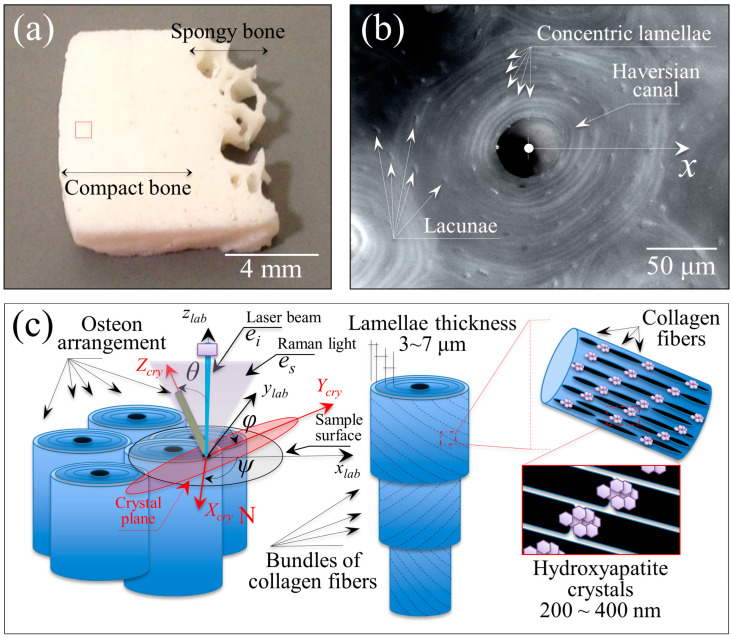
(**a**) Photograph of the human bone sample used in this research (measurement area in red square), (**b**) high magnification laser microscope image of a Haversian system, (**c**) model of the osteon arrangement, the lamellar structure, and the collagen fiber and hydroxyapatite crystal arrangement. The Greek letters *θ*, *φ*, and *ψ* represent three Euler angles, which describe the rotations of the Cartesian system linked to the hydroxyapatite crystals (*X_cry_*, *Y_cry_*, and *Z_cry_*) with respect to the laboratory axes (*x_lab_*, *y_lab_*, and *z_lab_*), while the polarization vectors of incoming and scattered light, referred to as *e_i_* and *e_s_*, respectively, are given by Equation (1).

Figure 1a,b shows the investigated sample taken from a human proximal femoral tissue (i.e., the shown sample surface is perpendicular to the femur long axis) and a laser micrograph of a single osteon from the same sample (boxed region in Figure 1a; imaged in the neighborhood of a central Haversian canal surrounded by osteocyte lacunae), respectively. In Figure 1c, a schematic draft of the hierarchical structure of osteons is given together with the definitions of the laboratory coordinate system and Euler angles. Both Cartesian and Euler coordinates serve to describe the position and orientation of the hydroxyapatite crystals in three dimensions. The polarization directions of incident and scattered light during Raman experiments are also represented. With reference to the draft in Figure 1c, we shall specifically focus here on the Raman determination of local orientation for the hydroxyapatite *c*-axis with respect to the main axis of the Haversian canal. We also assume that individual bundles of collagen fibrils fold around the Haversian canal according to a spiral configuration with approximately a constant angle with the Haversian canal axis. This information is then applied to discuss the pattern orientation of the contiguous collagen fibers constituting the individual (concentric) lamellae of the osteon. In polarized Raman experiments, we adopted the so-called Porto formalism, which employs two distinct systems of Cartesian axes associated with the incoming and the scattered radiation and described by a total of four rotational indexes. The formalism, expressed as *i*(*kl*)*j*, means that the incident light is propagated along the *i* direction with its electric vector in the *k* direction, while the Raman scattered light is collected from the *j* direction with the analyzer placed so that it passes light with the electric vector aligned along the *l* direction. In other words, the symbols outside and inside the brackets refer to the directions of light propagation and electric vector, respectively. The symbols *ei* and *es* in Figure 1c are the unit polarization vectors of the electric field for incident and scattered light, respectively. In all experiments described in the remainder of this paper, the polarization of the incident light was fixed (i.e., parallel to the y-axis), while both parallel and cross polarization were applied to the Raman scattered light in a backscattered geometry. Such configurations corresponded to $z(yy)\bar{z}$ and $z(xy)\bar{z}$, respectively (cf. Figure 1c for the choice of Cartesian axes and Euler angles). The configurations expressed as $z(yy)\bar{z}$ and $z(xy)\bar{z}$ will henceforth be simply denoted as the “parallel” and “cross” polarization configurations, respectively. The unit polarization vectors can be expressed in Cartesian coordinates, as follows:

(1) $$e_{i\;xyz}=\left(0\;1\;0\right),\;e_{s\;xyz}^{\left|\right|}=\left(\begin{array}{c} \;0\;\\ \;1\;\\ \;0\; \end{array}\right),\;e_{s\;xyz}^{⊥}=\left(\begin{array}{c} \;1\;\\ \;0\;\\ \;0\; \end{array}\right)$$

where the superscripts, ‖ and ⊥, refer to the parallel and cross configurations, respectively.

## 3. Results

**Figure 2:**
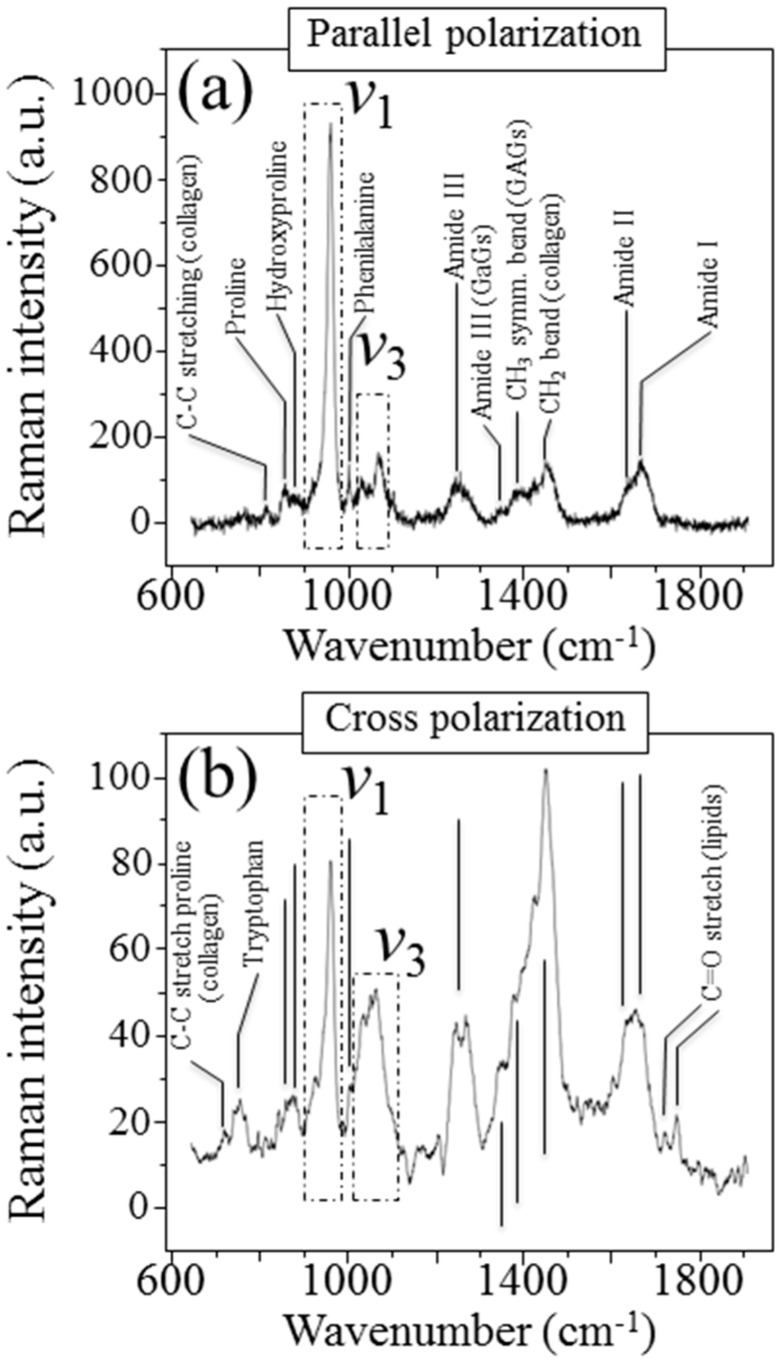
Representative Raman spectra of cortical bone sample in Figure 1, in (**a**) parallel ($z(yy)\bar{z}$)- and (**b**) cross ($z(xy)\bar{z}$)-polarized configurations. The spectra are representative of five spots (for each polarization geometry) collected with a 10× optical lens, which approximately covered the red-squared area in Figure 1a.

Figure 2a,b shows the parallel- and cross-polarized Raman spectra, respectively, of the cortical bone sample shown in Figure 1a. The labels locate specific vibrational bands from both hydroxyapatite (mineral) and collagen components. For the sake of completeness, we labeled all the observed bands in Figure 2. However, we will henceforth only focus on the mineral-related Raman bands. The apatite bands (ν_1_ and ν_3_) reflect the vibrational modes that have been defined for hydroxyapatite single crystals [15,16]. The Raman bands of organic origin include Amide I~III vibrations and basic C-C stretching in collagen and glycosaminoglycans (GAGs), CH_3_ and CH_2_ bending vibrations, and amino acids (phenilalanine, proline, and hydroxyproline) vibrations. Moreover, the cross polarization mode revealed additional bands related to tryptophan (at around 750 cm^−1^) and C=O stretching in lipids. The adoption of different polarization geometries significantly alters the relative intensity ratio of vibrational signals from the mineral phase and with respect to the amide bands of collagen.

**Figure 3:**
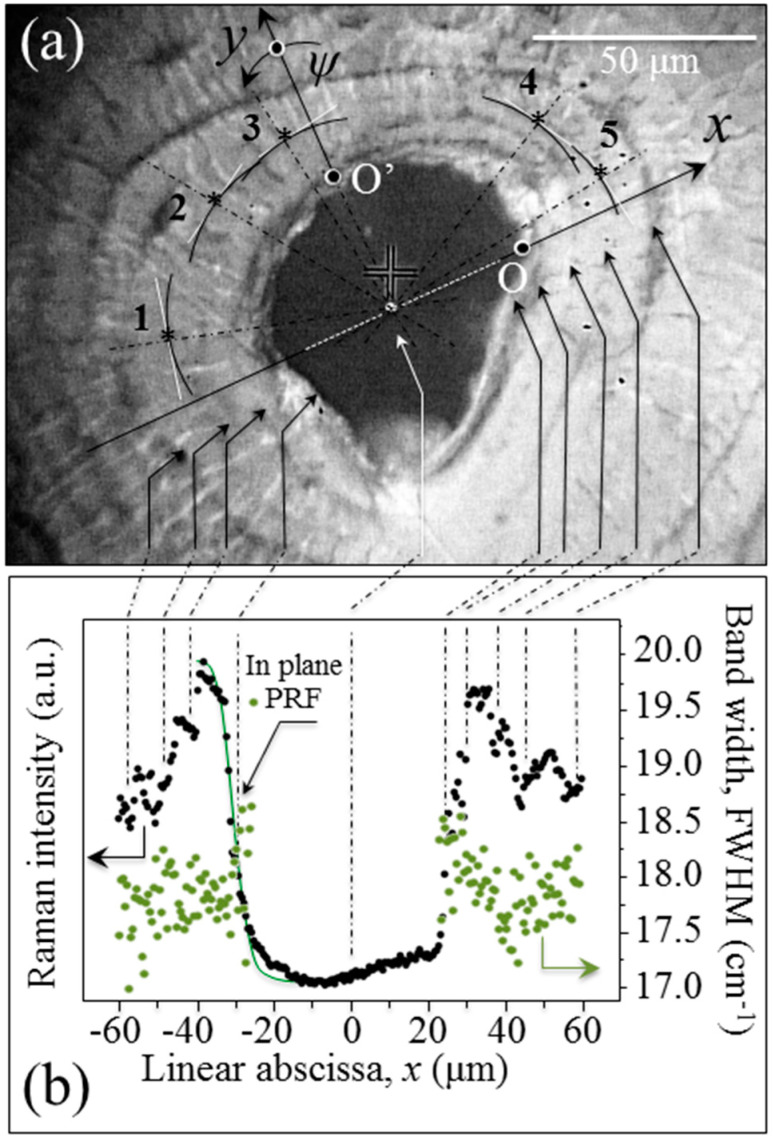
Osteon Raman cross-section in proximity to a Haversian canal, (**a**) optical image of the investigated area and (**b**) intensity and width of the Raman signal as a function of position along the line. Measurement locations are marked with an asterisk.

By applying spectroscopic methods previously developed for multi-crystalline materials [17], the intensity variations of the Raman bands originated from the mineral phase can be used to characterize the local orientation of the hydroxyapatite nano-sized crystals in the surroundings of the osteonal structure. We first performed non-polarized Raman analyses in the neighborhood of several Haversian channels in order to clarify the patterns of local orientation in bone apatite crystallites. Figure 3a is an optical micrograph showing one of the investigated osteonal areas in the neighborhood of a Haversian canal in human (femur) bone tissue, across from which a Raman line scan was performed (cf. linear abscissa, *x*). In Figure 3b, the results of Raman line scan are plotted in terms of scattered intensity and band width (full width at half maximum; FWHM) for the hydroxyapatite emission at around 960 cm^−1^ and related to the symmetric stretching, *ν*_1_, vibrational mode of the phosphate group.

The salient phenomenological features revealed by these experimental plots could be summarized as follows:

(i) Despite the irregular morphology and the asymmetrical shape of the channel, the intensity trend was symmetric and strongly related to the geometry of the Haversian channel itself. As an obvious consequence of the finite size of the Raman probe, when the probe approached the cavity, the Raman intensity exponentially decreased. The decreasing trend could be fitted according to a procedure explained in previous literature [15]. By doing so, the in-plane probe response function (PRF) could be determined (cf. full line in Figure 3b) as a Gaussian function, which revealed a probe diameter ~2.8 μm.
(ii) The Raman intensity experienced periodic fluctuations that could be related to the observed geometry of osteonal lamellae, as indicated by the arrows relating Figure 3a,b.
(iii) The Raman bandwidth, FWHM, of the hydroxyapatite band at ~960 cm^−1^ showed a relatively low scattering and no clear link to the geometry of the osteonal lamellae. However, an exception to this trend was the very first osteonal lamella from the side of the Haversian canal, which displayed a tendency to broaden the *ν*_1_ emission.

**Figure 4:**
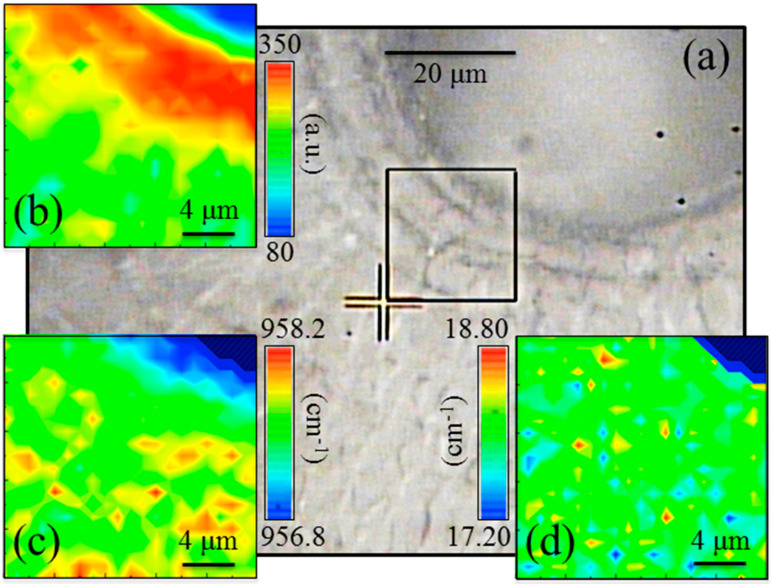
Localized Raman maps acquired in proximity of an Harvesian channel, (**a**) optical image of the surrounding region, (**b**) Raman intensity, (**c**) Raman band position, and (**d**) width of the band at about 960 cm^−1^ related to *ν*_1_ vibrations of PO_4_^3−^.

In order to give statistical validity and to provide a two-dimensional (2D) view of the line scan presented in Figure 3b, we carried out Raman mapping in the bone tissue surrounding the Haversian canals (cf. area in the squared inset to Figure 4a). Hyperspectral maps of Raman intensity, band spectral position, and FWHM are depicted in Figure 4b–d, respectively. The maps, which were collected with focusing on the sample-free surface and have a lateral resolution of 1 μm, confirmed the trends obtained with the line scan characterization with respect to Raman intensity and FWHM (cf. the above points (ii) and (iii) and the maps in Figure 4b,d, respectively). In addition, the 2D mapping revealed a systematic shift toward lower wavenumbers for the *ν*_1_ spectral position in the immediate neighborhood of the Haversian canal (cf. Figure 4c). Such morphological variation of the Raman band arises from a chemical perturbation of the apatite structure as previously observed in other apatite-based biomaterials [1,2,15,16].

**Figure 5:**
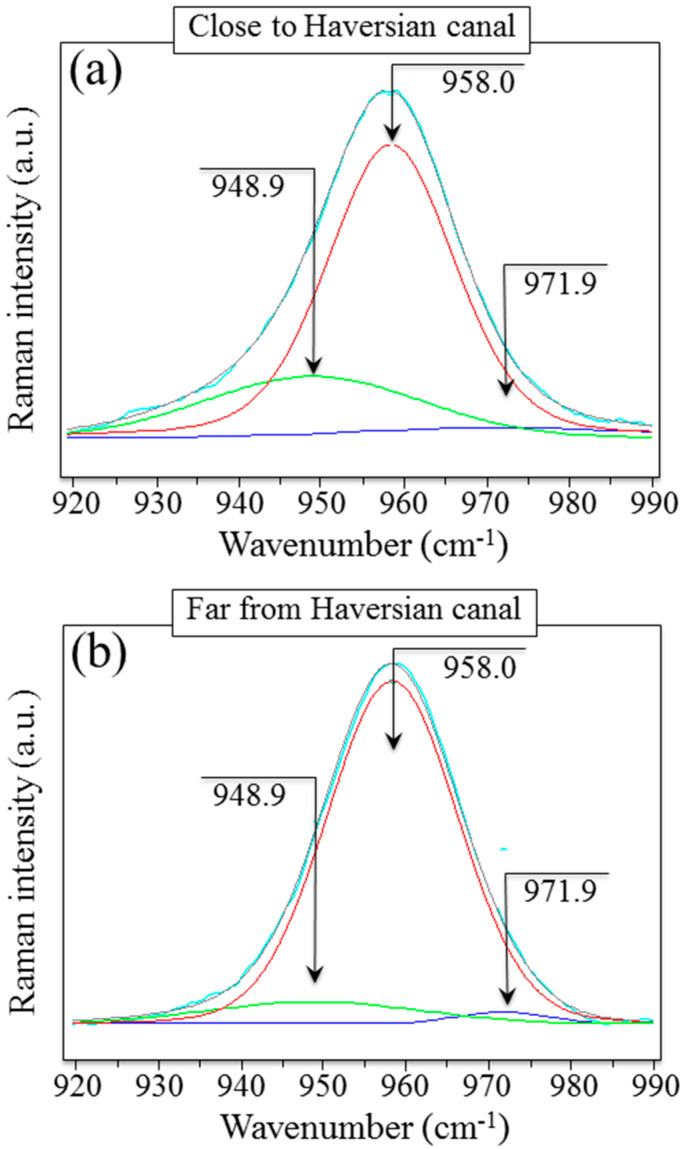
Representative Raman spectra in the region between 920 and 990 cm^−1^ taken in (**a**) proximity and (**b**) at a distance of about 80 μm from an Harvesian channel. The black line is the experimental signal; green, red, and blue lines represent deconvoluted Lorentzian–Gaussian sub-bands at 948.9, 958.0, and 971.9 cm^−1^, respectively; the light blue line is the sum of the three deconvoluted sub-bands. As seen, this latter band quite precisely overlaps the experimental signal.

Figure 5a,b shows a comparison between Raman scatters collected in proximity to and relatively far away (30 μm) from the Haversian canal, respectively. The related spectral deconvolutions (also shown in the respective figures) clarify the origin of the observed broadening. As compared to locations far away from the border of the Haversian canal, the overall Raman signal from the neighborhood of the Haversian canal broadens and shifts toward lower wavenumbers. Upon deconvolution, the main P-O stretching band component can be commonly located at 958.0 cm^−1^. However, a sub-band located at 948.9 cm^−1^ becomes ~2.7 times more intense close to the Haversian canal, which both broadens and shifts the overall emission in that spectral region.

A negligible sub-band contribution is also commonly found at 971.9 cm^−1^ both close to and far from the Haversian canal. This trend is similar to that observed in the case of demineralization of the enamel structure [1,15], in which a loss in uniformity of crystal orientation and altered stoichiometry of the main hydroxyapatite phase gives rise to locally altered structures, also including the formation of Ca vacancies. Poor crystallinity and imperfect stoichiometry in bony apatite indeed reflects the presence of vacancies in its lattice, which in turn leads to a reduction in crystal symmetry. Off-stoichiometry might arise from the presence of HPO_4_^2−^, CO_3_^2−^, Na^+^, and Mg^2+^ ions, either substituted for into the apatite lattice or chemically adsorbed onto its surface. From the vibrational physics viewpoint, the loss of screw-axis Ca^2+^ (i.e., Ca^2+^ vacancy formation) requires H+ proton balancing for guarantying electrical stability. Balance might be achieved either through the introduction of interstitial H in the apatite lattice or through the formation of H_2_O molecules, or both. The formation of interstitial H creates new binding bonds with the neighboring O ions, which in turn weakens the P-O bonding strength and decreases its vibrational energy. Accordingly, the observed spectral position of the P-O stretching (*ν*_1_) band shifts toward lower frequencies when interstitial hydrogen enters the hydroxyapatite lattice. The opposite trend is induced by water formation, with shifts toward higher frequencies of the P-O stretching band. Translating these notions into the interpretation of the band-shift trend shown in Figure 4b, one might conclude that the first lamella close to the Haversian canal is actually rich in substituted or chemically adsorbed foreign ions. It is thus rich in Ca^2+^ vacancies and undergoes lattice electrical compensation through the introduction of interstitial H ions. The high spectral resolution (±0.1 cm^−1^) with which the present Raman analyses were performed enabled us to reveal and make this trend measurable. While similar large-scale structural deformations can be easily detected even by low-resolution Raman spectroscopes and used for diagnostic purposes [17], such detections do not possess the resolution required for the detection of the crystallographic orientation.

A step forward in understanding the crystallographic arrangement of the apatite nano-sized crystals around the osteonal structure was taken by carrying out in-plane rotational experiments with a polarized Raman probe at selected locations, as labeled 1~5 in Figure 3a. In this experiment, the probed locations were selected within the same lamella and, conceivably, on the same spiral axis of collagen fibrils. Raman intensity data were also collected with the focal plane of the probe set at two different depths (i.e., *z* = 0 and 10 μm).

**Figure 6:**
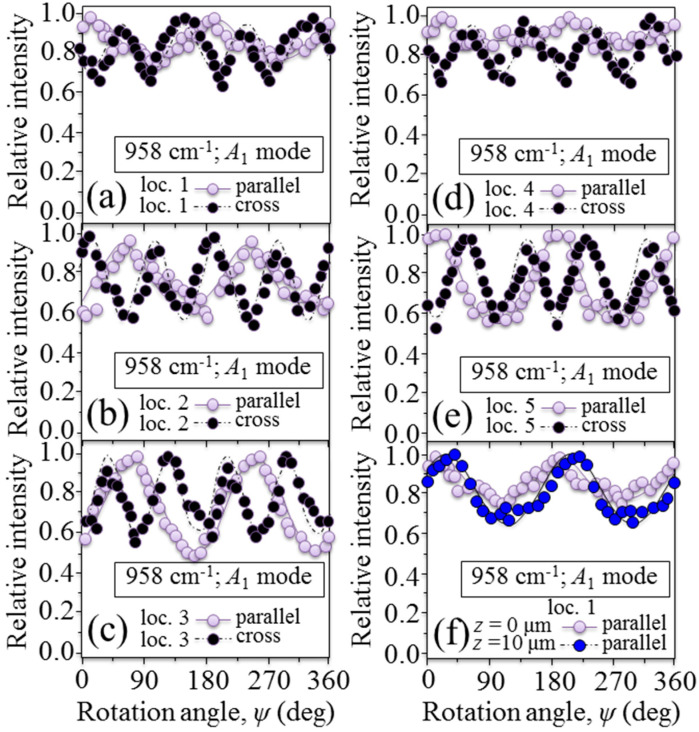
Angular dependence of the relative Raman intensity in parallel and cross light polarization for the band located at about 960 cm^−1^ and related to ν_1_ vibrations of PO_4_^3−^; (**a**–**e**) results obtained at five different locations and (**f**) effect of focusing depth on the relative Raman intensity in parallel polarization only; *ψ* is the in-plane Euler angle depicted in Figure 1c.

The results of the polarized Raman spectroscopic characterization are summarized in Figure 6a–e for cross and parallel trends at locations 1~5, respectively. All the above characterizations were performed with focusing the polarized Raman probe on the sample-free surface. As an example of the in-depth characterization carried out at each location, Figure 6f shows a comparison between the parallel rotational trends of Raman intensity recorded at *z* = 0 and 10 μm in correspondence to Location 1. Throughout all experiments, the Raman probe was kept in a constant confocal configuration and its in-depth size was ~10 μm. In all cases, we obtained sinusoidal functions for polarized Raman intensity trends as a function of the in-plane rotation angle, *ψ*, which proves the existence of a (average) preferential orientation axis for the nano-sized apatite crystallites. We fitted the sinusoidal dependencies according to the selection rules and Raman tensor elements by applying the ODF formalism [1].

The results of these calculations are shown in Table 1 in terms of average Euler angles and ODF Hermans’ parameters.

The salient outputs of the crystallographic data can be summarized as follows:

(i) The out-of-plane (with reference to the (*x_lab_*, *y_lab_*) plane of measurement) Euler angle, *θ*, (cf. its definition in Figure 1c) showed little variation within the interval 20~24° of inclination with respect to the main axis of the Haversian channel. This trend was the same for each probed location upon focusing the probe at the sample surface and in its sub-surface.
(ii) The in-plane (with reference to the (*x_lab_*, *y_lab_*) plane of measurement) Euler angle, *ψ*, (cf. its definition in Figure 1c) showed a clear and systematic variation following the spiral trajectory of the fibril axis. The trend was the same at the surface and in the depth of the sample, but there was an angular lag between axial orientations at different depths for the same location.
- The ODF characterization revealed a fairly high degree of orientation at any location both at the surface and in the depth of the sample. The primary Hermans’ parameter, *P*_2_<cos*β*>, showed values close to unity and only varied within the narrow interval 0.82~0.97.

The surprising regularity of the three-dimensional assembly and alignment of the nano-scale-sized apatite crystals is in agreement with a similar structural refinement recently reported in a polarized Raman study of the assembly of collagen fibrils [11]. Note that, while the apatite crystals result, on average, as parallel to the axis of the collagen fibrils, they follow a spiral-like pattern in their local arrangement along the fibril length.

**Figure 7:**
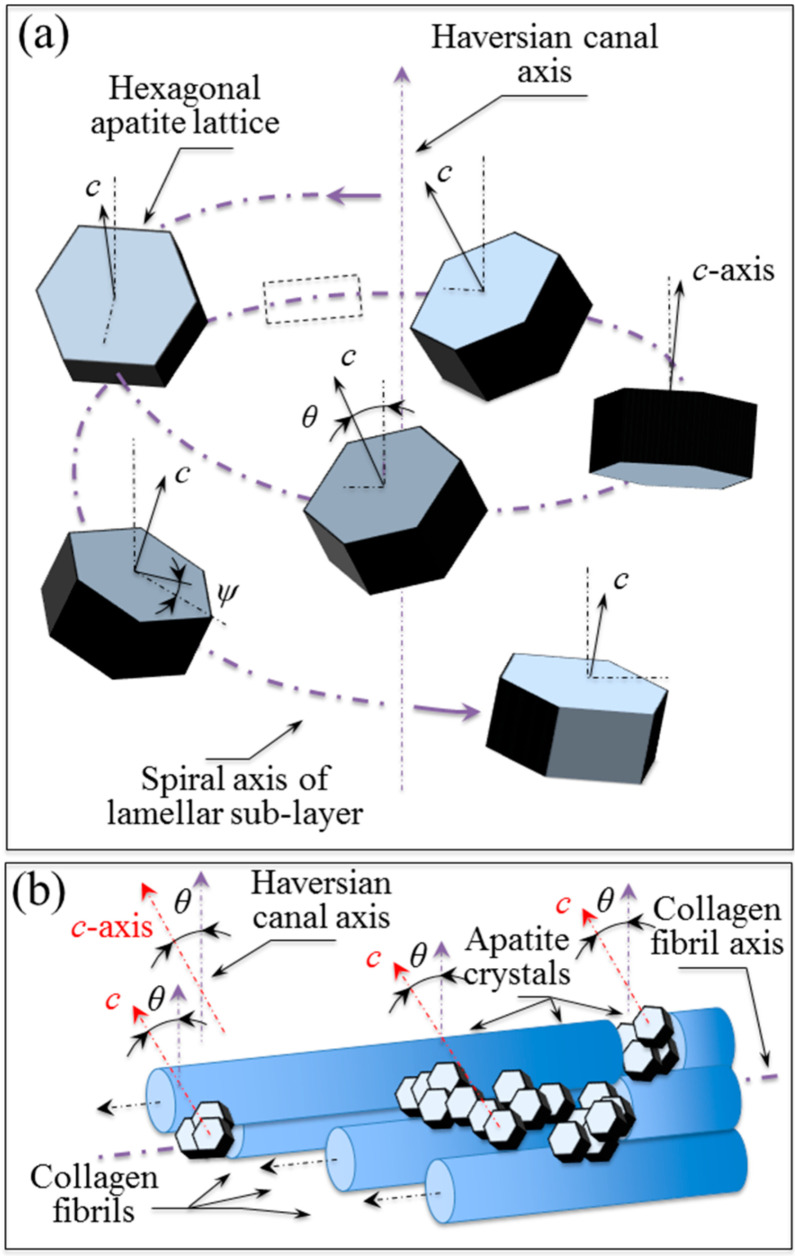
Representation of the average crystal orientation of the nano-sized apatite crystals within the collagen fibrils (**a**) along the spiral fibril axis trajectory, and (**b**) the relationship between the apatite crystal and fibril axis direction.

Complementary to that study, Figure 7 attempts to represent the average crystal orientation of the nano-sized apatite crystals comprised within the collagen fibrils along the spiral fibril axis trajectory (Figure 7a) and the relationship between the apatite crystal and the fibril axis directions (Figure 7b). If the angle comprised between individual spiral axes of collagen fibrils and the axis of the main Haversian canal remains constant, the angle between the average orientation of the apatite nano-sized crystallites and the collagen fibril axis could also be considered to be constant. Under this hypothesis, we can attempt to correlate the geometrical patterns, as postulated by other authors for collagen fibrils in the neighborhood of the osteonal structure, to the patterns of apatite crystal orientation observed in this study.

**Figure 8:**
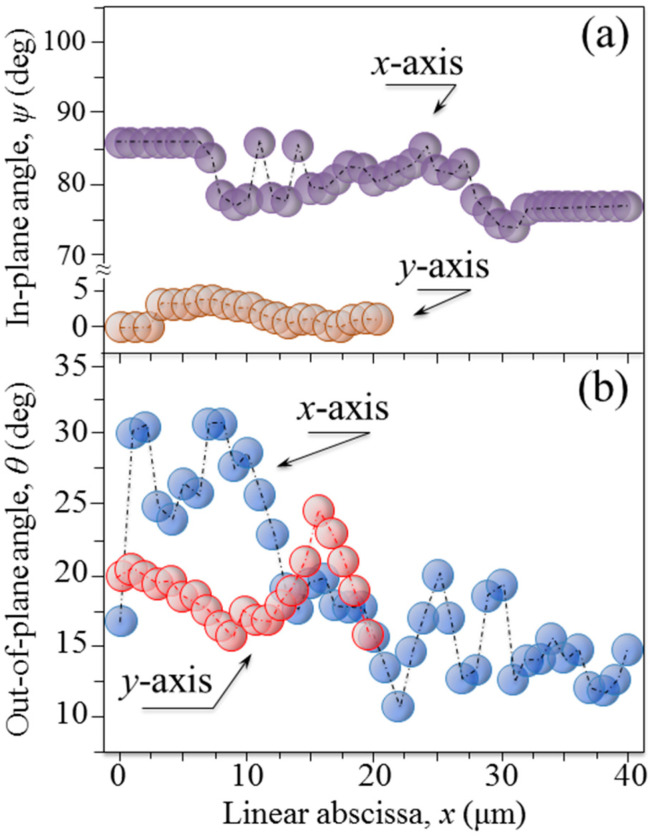
Plots of the observed trends of (**a**) in-plane, *ψ*, and (**b**) out-of-plane, *θ*, orientation angles of apatite crystals, respectively, as recorded along the *x*- and *y*-axis.

Figure 8a,b shows plots of the observed trends of in-plane, *ψ*, and out-of-plane, *θ*, orientation angles of apatite crystals, respectively, as recorded along the *x*- and *y*-axis (starting from the origin, O and O′, respectively) indicated in Figure 3a. The in-plane tilting angle, *ψ*, whose origin and versus are indicated in Figure 3a, varied within relatively narrow ranges of 76~86° and 0~5° for the *x*- and *y*-axis, respectively. Moreover, despite the narrow lateral step selected for the angular characterization (i.e., 1 μm), the polarized Raman probe could hardly resolve a clear periodic dependence for the in-plane *ψ* angle (Figure 8a). On the contrary, the variation of the out-of-plane tilting angle, *θ*, was more marked with an angular excursion of 10~30° and 16~26° for the *x*- and *y*-axis, respectively. Moreover, the angular periodicity could be related to the visible structure of the osteonal lamellae (Figure 8b). Considering the finite size of the Raman probe, which requires a deconvolutive procedure, we hereafter attempt to rationalize the observed pattern of out-of-plane tilt according to previously proposed models for collagen fibrils [11].

## 4. Discussion

**Figure 9:**
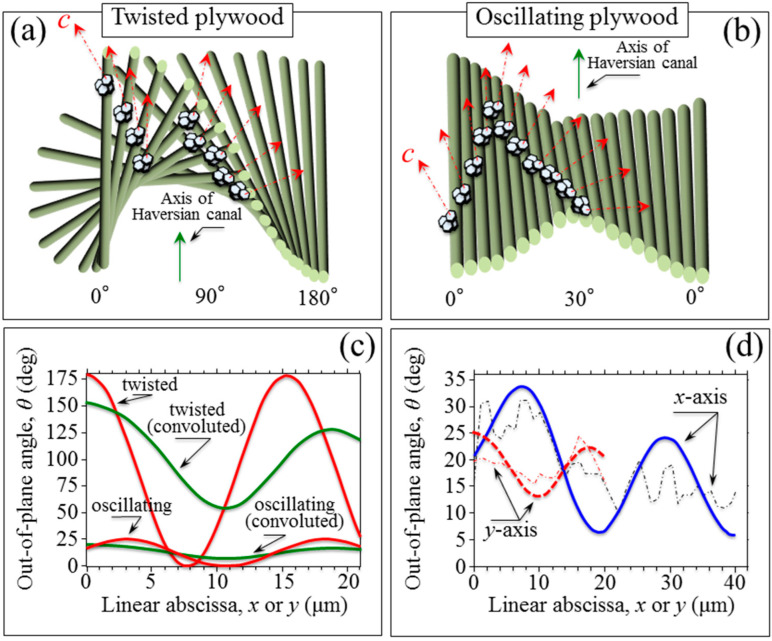
(**a**) Twisted and (**b**) oscillating plywood theoretical structure for hydroxyapatite and collagen fibrils, theoretical trend for the out-of-plane angle *θ* as function of the linear abscissa for (**c**) twisted and (**d**) oscillating plywood, respectively.

According to periodic variations of the intensity of Raman signals from collagen fibrils, as collected on human (proximal femur) bone samples, two different collagen fibril arrangements within lamellae were proposed: the twisted and the oscillating plywood pattern. [11,18,19] Evidence for a coexistence of both plywood patterns in the same osteon was also provided, thus validating earlier findings [20,21,22,23,24,25]. The experimental data collected in this study can be interpreted with reference to the above two stereological fibril arrangements under the hypothesis that the tangent at each point of the spiral axis (i.e., for each individual collagen fibril) maintains constant angles with both radial and spiral axes (i.e., the perpendicular and parallel directions to the Haversian canal, respectively; cf. drafts in Figure 9a,b for twisted and oscillating plywood, respectively) [26,27]. In other words, one should assume that each individual collagen fibril obeys the trajectory of an equiangular (or self-similar) spiral. The dependence of the in-plane angle, *θ*, shown in Table 1, indeed seems to suggest that the above hypothesis is conceivable regarding the misalignment of the radial direction. As the in-plane angular orientation of the apatite *c*-axis along an individual fibril spiral is found to approximately follow a regular variation with respect to a pre-selected in-plane *y*-axis direction according to an anti-clockwise rotation (cf. locations 1~5 and orientation labels in Figure 3a). Based on this encouraging output, we attempted a spatial deconvolution of the out-of-plane angle, *θ*, along the two radial directions, *x* and *y* (cf. labels in Figure 3a and data in Figure 8b). Since the *θ* values retrieved at a fixed location along the *z*-axis (i.e., the axis perpendicular to the sample-free surface) showed a nearly constant value, we neglected variations along the in-depth direction in deconvoluting the Raman probe. We then implemented an in-plane probe deconvolutive routine according to the following equation:

(2) $$\bar{\theta }\left(x_{0},y_{0}\right)=\frac{\int_{0}^{\infty }\int_{0}^{\infty }\theta \left(x,y\right)\;exp\left[-2\frac{{\left(x-x_{0}\right)}^{2}+{\left(y-y_{0}\right)}^{2}}{R^{2}}\right]dx\;dy}{\int_{0}^{\infty }exp\left[-2\frac{{\left(x-x_{0}\right)}^{2}+{\left(y-y_{0}\right)}^{2}}{R^{2}}\right]dx\;dy}$$

where $\bar{\theta }\left(x_{0},y_{0}\right)$ is the observed (probe convoluted) angular trend at a given location $\left(x_{0},y_{0}\right)$, and θ(x,y) is the angular distribution within the Raman probe. This latter function was set as a linear combination of twisted and oscillating plywood patterns, as follows:

(3) $$\begin{array}{c} \theta \left(x\;or\;y\right)=V_{t}\times \theta_{t}\left(x\;or\;y\right)+\left(1-V_{t}\right)\times \theta_{0}\left(x\;or\;y\right)\\ =\left\{h_{t}\times \exp\left[-g_{t}\times \left(x\;or\;y\right)\right]\right\}\times \left\{A_{t}\cos\left[B_{t}\times \left(x\;or\;y\right)+D_{t}\right]\right\}\\ +\left\{1-h_{t}\times \exp\left[-g_{t}\times \left(x\;or\;y\right)\right]\right\}\times \left\{A_{0}\cos\left[B_{0}\times \left(x\;or\;y\right)+C_{0}\right]+D_{0}\right\} \end{array}$$

where $V_{t}$ and $\left(1-V_{t}\right)$ represent the volume fractions of twisted and oscillating plywood units within the Raman probe, respectively, and $\theta_{t}$ and $\theta_{0}$ are the (constant) out-of-plane tilting angles in twisted and oscillating plywood, respectively. The symbols $h_{t}$, $g_{t}$, $A_{t}$, $B_{t}$, $C_{t}$, $D_{t}$, $A_{0}$, $B_{0}$, $C_{0}$, and $D_{0}$ represent constant parameters, which were determined by best fitting the experimental data. The former two parameters serve to identify the volume fraction function, $V_{t}\left(x\;or\;y\right)$ of twisted plywood structures.

Figure 9c shows the theoretical trends for both *θ* and *ψ* angles along a radial direction with respect to the main axis of the Haversian canal. Plots are given for both twisted and oscillating plywood patterns, together with their respective convoluted trends according to Equations (2) and (3). In Figure 9d, the experimental *θ* trends obtained as functions of the abscissas *x* or *y* (cf. Figure 3a) are replotted from Figure 8b and best fitted by means of the convoluted curves according to Equations (2) and (3). As seen, it is possible to interpret the obtained data as a convolution of twisted and oscillating plywood nano-sided structures [28,29]. However, unlike a previously published study on collagen fibrils, the volume fraction of twisted plywood units was not found to be the predominant one. Moreover, it was hard to find a repetitive trend on different line scans at different osteonal sites as a function of distance from the Haversian canal. The lack of a general equation for theoretical predictions might certainly be associated with the limited spatial resolution of the Raman probe, but it might also reflect the presence of a fraction of disordered (random) collagen fibrils within the lamellar organization [30,31].

Such presence somewhat “scrambles” the structural order on a longer range. Although ODF-empowered selection rule-based algorithms for hydroxyapatite crystals have now become available for three-dimensional quantifications of orientation patterns in natural biomaterials, clarification of the structural assembly at osteonal sites appears to be one of those cases that is still awaiting future improvements in spatial resolution of the Raman probe. Nevertheless, the Raman methodology here proved to be capable to concurrently provide insights into the relationship between the chemical composition and structural arrangement in cortical bone at a sub-micron scale. Complementing current diagnostic tools, polarized Raman spectroscopy could thus contribute to elucidate the causes of observed mismatches in functionality between healthy and diseased bone tissue at the molecular scale.

## 5. Conclusions

Raman spectroscopy proved to be a powerful tool for structural analyses of bone, not just for qualitative analyses of constituents, but also to obtain information of crystallographic orientation and molecular structure, provided that a polarized probe with resolution enhanced to a sub-micrometric scale by PRF algorithms is adopted.

It was observed that the plywood structure of collagen and apatite can be effectively studied by Raman spectroscopy, resulting in geometrical parameters associated with both the collagen fibril and the hydroxyapatite crystal orientations in a three-dimensional space.

The standardized procedure proposed in this research can be extended to tissues affected by a variety of diseases, such as osteoporosis, osteonecrosis, osteopetrosis, osteogenesis imperfecta, and osteomalacia. Correlations between spectroscopic structural parameters and other conventional diagnostic methods might then be used for early detection of osteopathic diseases.

## Figures and Tables

**Table 1:** Average Euler angles and ODF Hermans’ parameters as obtained at the five different locations of Figure 3a.

Location.	Depth, *z* (μm)	*P*_2_<cos*β*>	*P*_4_<cos*β*>	*A*	*λ*_2_	*λ*_4_	*θ*	ψ
Point 1	0	0.97	0.9	2.35 × 10^−7^	−7	−8.5	20.87	70
	10	0.95	0.86	6.76 × 10^−5^	−2.5	−7	37	63.05
Point 2	0	0.94	0.82	1.91 × 10^−5^	−6.5	−4	23.68	25.89
	10	0.96	0.88	3.17 × 10^−7^	−9	−6	20.63	47.48
Point 3	0	0.82	0.53	5.21 × 10^−4^	−6	0	20.6	13.77
	10	0.87	0.7	1.04 × 10^−3^	−3	−3	24.22	4.68
Point 4	0	0.95	0.85	8.27 × 10^−6^	−6.5	−5	19.64	−73.2
	10	0.96	0.87	4.71 × 10^−7^	−9.5	−5	20.79	−73.4
Point 5	0	0.92	0.78	3.15 × 10^−4^	−3.5	−4	20.02	−81.01
	10	0.91	0.77	4.98 × 10^−4^	−3	−4	21.35	−81.27

## Data Availability

Data is available upon reasonable request to the corresponding author.
